# Appendicitis
1Read at the Meeting of the Bath and Bristol Branch of the British Medical Association, on January 31st, 1894. A Report of the Discussion Which Followed is Given under "Meetings of Societies."


**Published:** 1894-03

**Authors:** James Swain

**Affiliations:** Assistant-Surgeon to the Bristol Royal Infirmary


### APPENDICITIS.[1]

BY

JAMES SWAIN, M.D., M.S. Lond., F.R.C.S. Eng.,

*Assistant-Surgeon to the Bristol Royal Infirmary.*

---

WHEN we consider that about 20 per cent. of all bodies submitted to *post-mortem* examination, other than those which have died from appendicitis, show evidences of former appendicular lesions, and when we reflect on the serious clinical phenomena associated with the disease which forms the subject chosen for discussion to-night, its great importance, both to the physician and surgeon, becomes at once apparent.

The subject is, however, a large one, and I shall not therefore discuss the relationship of appendicitis to perityphlitis and other similar conditions, except so far as they depend on a primary inflammation in the vermiform appendix itself. This point has, to my mind, been so abundantly proved of late years that I will not now enter upon it, merely contenting myself by calling to mind that, according to Fergusson, in about 77 out of 200 normal subjects—that is, about one-third—the appendix

[1] Read at the meeting of the Bath and Bristol Branch of the British Medical Association, on January 31st, 1894. A report of the discussion which followed is given under "Meetings of Societies."

was in close relation to the sub-peritoneal tissue; so that, had it become inflamed and perforated, the abscess would probably be situated in the cellular tissue around the cæcum and in the iliac fossa. In other words, the condition of paratyphlitis so produced would really be due to appendicitis. The chief points which I think may be most profitably considered, are:

(1) The causation of the disease.

(2) Some classification on a pathologico-clinical basis, and the chief symptoms to which each of these varieties may give rise; with a few suggestions as to differential diagnosis.

(3) The question of treatment.

First, then, with regard to etiology. According to Talamon,[1] some kind of foreign body has been noted in 60 per cent. of cases of appendicitis. Pins, fish-bones, biliary calculi, and vegetable seeds have been described, but fæcal concretions are by far the most common, forming about 50 per cent. of all cases; and in order to account for those cases in which no foreign body has been found, it has been suggested that the scybalon has been dislodged, dissolved, or lost.

The frequent existence of fæcal concretions will, I think, justify us in considering where such concretions are formed, and how they act in producing inflammation of the appendix.

The generally accepted idea, according to Talamon, is that rounded fæcal pellets are formed in the cæcum, and that one or more of such scybala find their way into the appendix and there set up a spasm and inflammation of that tube. Considering that Dr. Joseph Bryant[2] found fæcal matter in the appendix in about 45 per cent. of a series of *post-mortem* examinations on persons who had died from other causes than appendicitis, I think it more likely that the hardened fæcal mass is formed in the appendix itself by the inspissation of such fæcal matter as may be considered to be normally present there. There are two points in favour of this view: firstly, the shape of the concretion; and secondly, the existence of a valve, described by Gerlach, at the orifice of the appendicular canal.

[1] *Appendicitis and Typhlitis*, 1893. [2] *Ann. Surg.*, vol. xvii., 1893, p. 164.

As regards shape, the fæcal mass has in my experience generally taken an elongated form, as if moulded in a tube,—resembling a small date-stone, rather than being rounded as ordinarily described. On this matter I should much like an expression of opinion of the members of this Association, as the accumulated experience would, I think, help to decide the question at issue.

With reference to the so-called valve, I have, after careful examination, come to the same conclusion as Professor Struthers, that it is simply one of those crescentic folds that always occur when one tube joins another at an acute angle; but yet this is sufficient to obstruct the entrance of a fæcal concretion from the cæcum, although, as we have seen, it practically offers no resistance to the entrance of ordinarily soft fæcal matter.

Granting then the presence of a fæcal concretion, it is supposed that the irritation thereof produces a spasm of the muscular tissue of the appendix, analogous to the spasm produced by a biliary or renal calculus. That this spasmodic effort to expel the foreign body actually occurs has been shown by Morris[2] of New York, who has recorded a case in which he positively saw the appendix in a state of spasm, which subsequently relaxed. There is no doubt that these spasmodic attacks are the cause of the severe pain which occurs in the early stage of the disease, and this is supported by an interesting case, which came under my care last year, of a man who for eight months had been subject to attacks resembling those of relapsing appendicitis, and which had prevented him from doing his work for more than a week or two at a time during that period. I removed the vermiform appendix, which was quite free from those adhesions which are usually present in these cases, and on opening it I found it nearly full of semi-solid fæcal matter, but with practically no inflammation in its wall. The man has now been working for some months without any pain or inconvenience. Here, then, was a case in which the symptoms were apparently due to spasm only, for there was no

[1] *Edinb. M. J.*, Oct., 1893.
[2] *Ann. Surg.*, vol. xviii., 1893, p. 370.

chronic inflammatory thickening of the walls of the appendix and no peritonitis.

The spasm produced by the irritation of the foreign body causes compression of the walls of the canal and interference with their vascular supply; and then, if the irritation be sufficiently severe and prolonged, an inflammation—plastic, suppurative, or gangrenous—ensues.

Treves[1] thinks that the morbid changes are caused by torsion of the appendix, which thus produces compression of its walls, and distension with mucus of the distal portion, with ulceration or gangrene there, or at the seat of the bend. This theory will certainly account not only for cases where a fæcal concretion is formed, but also for cases where no such concretion exists; and considering that fæcal matter and mucus are found in normal appendices even more often than in cases of appendicitis, we are, I think, justified in assuming that the mere presence of fæces or mucus—both of them normal contents of the intestinal canal—would scarcely be likely to give rise to mischief, unless by their retention the appendix becomes over-distended by an accumulation of its secretions, or its walls become irritated by the hardening of the retained fæces. To this, however, I shall presently return; and although it is difficult to determine whether the torsion is primary and causes the peritonitis, or whether it is secondary to an antecedent peritonitis producing adhesion of the mesentery and kinking of the tube, we must admit that this condition of torsion could either produce, or exaggerate, the symptoms; and certainly in a large percentage of Treves's cases, torsion of the tube appears to have been present.

Why the fæcal material should become inspissated it is difficult to say, but we can readily imagine that an increase in the curvature of the appendix—which is normally due to its short mesentery—would virtually lead to the shutting off from the intestine of fæcal matter in the appendicular canal, and that this would be followed by absorption of fluid and hardening of the fæces. Indeed, it seems to me that the hardening of fæcal matter in the appendix might easily result from the

[1] *Surgical Treatment of Typhlitis*, 1890.

prolonged continuance of fæces in the canal, without any kinking of the tube whatever.

In a similar manner a real or apparent stricture of the appendix—the result of former ulceration or kinking—might give rise to retention of mucus, with distension of the tube and secondary inflammation, as I have already indicated.

There are two important conditions which we must consider in relation to the theory of concretion as a cause of appendicitis: one is that the disease is four times more common in males than in females, and the other is that about 76 per cent. of all cases occur below the age of 30. Can we account for these facts on the supposition that the symptoms are due to the formation of a foreign body in the appendicular canal?

With regard to the predominance of the male sex, I think the observations of Dr. Bryant[1] are important. He has shown that the greater the diameter of the appendix, within apparently normal limits, the greater the probability of the presence within it of fæcal or other foreign matter—so that in appendices $\frac{5}{16}$ of an inch in diameter, no less than 89 per cent. contained fæcal or other material; that in 70 per cent. of males and only 56 per cent. of females the appendix contained abnormal matter of some kind; and that the average diameter of the appendix of the male is about $\frac{1}{10}$ of an inch greater than that of the female. If we accept the theory of fæcal concretion as a causative agent, I think these observations go a long way towards explaining the fact that disease of the appendix is so much more frequent in males than in females. The common occurrence of the disease in early life is not so easily explained, as with the exception of the researches of Professor Struthers, I am not aware of any statement with reference to the calibre of the appendix at different ages. This is a subject that requires further elucidation; but so far as records go, it appears that the diameter of the appendix is relatively greater in the fœtus, and that in later life it is diminished. The evidence, however, is too scanty for us to draw any very definite conclusions; although, perhaps, it is slightly in favour of the theory I have been endeavouring to establish. Injury, cold, fatigue,

[1] *Loc. cit.*

and indigestion have been assigned as causes, and they probably act injuriously by inducing an increased peristalsis and spasm of the tube. I have never seen any other substances in the appendix except fæces, mucus, and pus; but bodies foreign to the intestinal tract, which form only a small percentage of cases, would act in the manner already described.

At any rate, I think we are justified in saying that the presence of a foreign body—fæcal or otherwise—is the exciting cause of the mischief in the great majority of cases. Many observers, however, are of opinion that in addition to the presence of the foreign body there is, in consequence of the irritation so produced, an active development of various bacteria, which then attack the tissues already diminished in vitality by the compression of the walls of the appendix. On this matter I have no personal experience whatever; but as the subject has lately received much attention, I do not like to leave it unnoticed as one of the possible factors in the inflammation, ulceration, or perforation of the appendix. Many organisms are found in the alimentary canal of normal individuals, but the one which has been specially credited with such lesions as that now under consideration is the bacillus coli communis—an organism which is identical with the bacillus pyogenes of urinary infection and with other forms described by various authors. According to Roswell Park, "this organism, which is constantly present in the intestinal canal, is not always a harmless inhabitant, but becomes at times an active invader. It does not confine itself alone to the intestinal mucosa, where it may set up most active desquamative lesions, but may pass this barrier and penetrate into the general circulation and exercise pernicious activity in numerous other organs, with toxic effects upon the system at large."[1] The causes of this increased virulence have not yet been ascertained. Macaigne[2] regards this bacillus as the cause of various forms of infectious enteritis, and believes that probably most, if not all, cases of appendicitis come also under this head. It is in cases of perforative peritonitis that the bacillus coli communis is most often found,

[1] *Ann. Surg*,, vol. xviii., 1893, p. 307.

[2] Quoted by Roswell Park.

—sometimes alone, sometimes with other organisms,—although perforation does not appear to be essential to its presence in the inflammatory exudation. One argument regarded as favouring the agency of some such organism is that the perforation in cases of appendicitis is not always at the point of contact of the foreign body, which would probably be the case if the perforation were due to compression of the walls of the tube at that point. On the bacillary theory it is supposed that the distension, spasm, or twisting of the tube already referred to brings about an obliteration of the single artery by which the appendix is usually supplied, and that the walls being thereby lowered in vitality will, when invaded by this organism, give way at that point which is most susceptible to that invasion—*i.e.*, at a spot where the vitality of the tube is most diminished, whether that spot be opposite to the position of the foreign body or not.

We have now to consider briefly the various pathological and clinical aspects of appendicitis. In order that we may not go too far afield, I shall omit all specific forms of appendicitis, such as that occurring in tubercular, dysenteric, or typhoidal ulceration of the intestines. In these cases the appendicular disease is only a part of a more general process, by which, however, the symptoms are necessarily somewhat obscured.

The first variety, and the least fatal, may be called simple appendicitis, by which I mean an inflammation of the walls of the appendix which does not extend to the peritoneum. The depth to which such inflammation may affect the walls of the appendix will of course vary; but in order not to confuse the issue, I shall not refer to the various sub-divisions which some observers have described. The symptoms are mainly those of colic, with a fixed pain somewhere about a point midway between the right anterior superior iliac spine and the umbilicus—McBurney's point,—a slight febrile condition, constipation and vomiting. These symptoms, which commence more or less suddenly, may at first be associated with general abdominal pains, which gradually become localised in the spot referred to.

The abdominal muscles are tense on the right side, and there is marked tenderness in the neighbourhood of McBurney's spot, where perhaps an enlarged appendix may be felt after the rigidity of the muscles has passed away. The symptoms usually subside in the course of seven or eight days. The slight degree to which the appendix may be pathologically affected, the marked presence of appendicular colic, and an occasional tendency to relapse are all shown by the case which I quoted when discussing the etiology of the disease, and Dr. Bull[1] has reported two similar cases.

The next variety—that of plastic appendicitis—is associated pathologically with a fibrinous inflammation of the neighbouring peritoneum, compared by Talamon[2] to the plastic pleuritis which may occur in cases of pneumonia. This plastic appendicitis is the ordinary non-suppurative, acute appendicitis. It commences with symptoms resembling those of the simple variety, but proportionately more severe, and is attended by the inflammatory peritoneal exudation referred to. This localised peritonitis is manifested by the presence, about the second day of the disease, of a more or less defined swelling in the right iliac fossa, varying in size with the extent of the peritoneum affected, but reaching sometimes as high as the umbilicus or extending downwards into the true pelvis.

In an ordinary case this exudation may be absorbed in about three weeks; but I have known it to continue for nearly double that length of time.

The general tendency is, however, towards recovery sooner or later.

The third variety—suppurative appendicitis—I wish to reserve, for reasons which will presently appear, for those cases of appendicitis associated with a *circumscribed* purulent peritonitis. In so far as the accompanying peritonitis is limited we have a pathological condition which resembles the plastic appendicitis just described; but without going into details, I may state that the balance of evidence is in favour of the suppurative form being caused by a perforation of the appendix, and the plastic form being unassociated with any such

[1] *Med. Rec.*, Ap. 20, 1890. [2] *Op. cit.*

lesion. Clinically, at all events, we may regard them as distinct varieties. The diffuse colicky pains, gradually limiting themselves to the right iliac fossa, with signs of localised peritonitis and tumefaction, resemble the symptoms of the plastic variety, but the temperature is apt to be rather higher—say 102° or 103° F. A fluctuating purulent swelling has sometimes been detected as early as the second day, but at other times not till the end of six or seven days or more. The tenderness on pressure and the rigidity of the muscles help to obscure the diagnosis, and we may have to rely for our belief in the existence of suppuration upon the general condition of the patient as to sweating and temperature, and upon a continuance of the illness beyond the time at which we might expect an amelioration in a more simple case. An examination of the rectum (and vagina in the female) in these cases is very important, as it may furnish most trustworthy information.

In one case I was able to diagnose the presence of pus by this means, when from the swelling of the abdomen it could not be detected; and in another case, in which suppuration was suspected, I felt justified, after a careful exploration of the rectum, in postponing any operative procedure, and the patient —a medical man—made an uninterrupted recovery.

Unlike the plastic exudation, this collection of pus shows no tendency to absorption, but if left to itself ends by bursting into the general peritoneal cavity, and thus causes a diffuse peritonitis which terminates fatally; or it bursts externally, or into one of the viscera, and may thus lead to recovery.

I must not leave the subject of suppurative appendicitis without referring briefly to that latent form of the disease which is generally associated with suppuration in the cellular tissue behind the peritoneum in the iliac fossa. The gravity of this form is only second to that of the rapidly perforative form which I shall presently describe. It owes its great fatality to its insidiousness, for it most often occurs after convalescence from acute diseases and in old age, with perhaps few other symptoms than vague colicky pains and a slightly elevated temperature, so that the discovery of suppuration emanating from a perforated appendix does not take place until the area

3

affected is so extensive that the damage is irreparable; or perhaps the sudden exacerbation of symptoms which first warns the physician of the gravity of his case is rapidly followed by a fatal issue.

The next variety—that of rapidly perforative or fulminating appendicitis—is more common in young people, and is the most fatal of any form of appendicitis. Its seriousness is shown by the fact that in at least 75 per cent. of perforative cases it was the *first* attack which was accompanied by the perforation. The strangulation of the appendix in the way already explained is most complete, and rapidly runs on to gangrene of its walls, which then become perforated, with the rapid diffusion of the septic contents over the peritoneal cavity. Perforation does not usually occur until the second or third day, being preceded by the general and local pains and vomiting, as in other varieties. The temperature is not at first much raised. With the onset of perforation the symptoms assume all the gravity of an acute general peritonitis. The pain, especially in the right iliac fossa, is more intense and rapidly spreads over the whole abdomen, the vomiting becomes incessant, constipation is practically absolute, and the pulse is small and frequent. The general symptoms are at first those of shock, and the temperature may be low, although it subsequently rises to 102° or more if the patient lives for any length of time. The abdomen is at first retracted and the abdominal muscles very tense, but later on there may be general abdominal distension from paralysis of the intestines. The face bears the usual anxious expression of acute abdominal disease. The patient may die in a day or two, apparently from a general septic condition, before much suppuration has occurred; in some cases he may drag on for a fortnight or more, but eventually he dies of a general suppurative peritonitis. According to Fitz,[1] 98 out of 176 cases died in the first week.

The last class which I shall mention is that of relapsing appendicitis. It is necessary to insist upon a clinical distinction

[1] *Am. J. M. Sc.*, vol. xcii., 1886, p. 321.

between recurrent cases, in which there is a definite return to health of some duration between the attacks, and relapsing appendicitis, in which the patient is practically never entirely free from symptoms between the exacerbations of the disease. It is to the latter that I now refer. Pathologically, relapsing appendicitis resembles the plastic form of the disease, but its definite clinical course makes it desirable to place it in a different category. Relapsing appendicitis is more often associated with the retention of mucus or pus, rather than fæcal matter, in the tube; but however that may be, we may suppose that the repetition of the attacks is simply due to a continuance of those local conditions which formed the starting-point of the disease. It is in this form, too, that Treves[1] has so frequently found the kinking of the appendix which he has described. The repeated attacks of peritonitis are, no doubt, protective, and hence we find that this variety is less fatal than any except the simple form. The disease may indeed actually cure itself by producing an obliteration of the appendix, and when we interfere we do so more often on account of its morbidity than its mortality.

The symptoms of the relapse are generally the same as the simple or plastic forms of the disease, and I shall not further describe them. Only rarely is the attack associated with suppuration. In about a week, when the acute stage is over, the patient begins to recover, but he does not do so completely. In this intermediate period he suffers from a feeling of uneasiness in the right iliac fossa, constipation or diarrhœa, abdominal distension, and indigestion. These symptoms get worse after several attacks, which vary in frequency and intensity, and the patient becomes a chronic invalid. In the intervals tenderness can generally be elicited near McBurney's point, and oftentimes the enlarged appendix can be felt.

These, then, are the main clinical aspects presented by cases of appendicitis. The most important questions to decide are, whether the appendix has become or is becoming rapidly perforated, and whether suppuration has occurred. I cannot now pursue these questions further than I have done in my

[1] *Op. cit.*

account of the various symptoms, but I hope that those of more extended experience will help to elucidate these very important considerations.

As regards general diagnosis, it is not surprising that the peculiar colicky nature of the pain in the early stage of appendicitis should lead to its confusion with such diseases as renal and hepatic colic. We must, however, be guided by the history of the case and the accompanying symptoms, and remember that the inflamed appendix almost always gives rise to a fixed painful spot. This fixed pain appears within 24 hours in about 72 per cent. of cases, and becomes localised in the right iliac fossa in 54 per cent. of all cases—corresponding practically with McBurney's point. But the position of the appendix is variable: it has been found under the liver, in a hernial sac, and in other unusual positions; so too much reliance must not be placed on the actual locality of the fixed pain. All that we can say is, that the point of greatest tenderness corresponds with the position of the inflamed appendix.

This variability of position also explains why we may be called upon to differentiate between appendicitis and diseases of the uterine appendages or perinephric abscesses.

The peritonitis and the obstinate constipation, distension, and vomiting caused by the disease have not infrequently led to a diagnosis of intestinal obstruction. We now know, however, that in all cases of acute peritonitis occurring in children and young adults, a perforative appendicitis must be thought of as a very probable cause of the mischief. In a case which was sent to me as an intussusception, the bulging which was felt *per rectum* was caused by a collection of pus in the neighbourhood of a perforated appendix.

From typhoid fever, appendicitis will best be differentiated by remembering the peculiar character of the pain and its more or less sudden onset, and by watching the course of the temperature and the accompanying general symptoms.

We now come to the question of treatment, the general principles being easily deduced from the clinical and pathological conditions which we have just considered. The American surgeons, regarding the probably infectious nature of the disease and the impossibility of stating that any individual case will not become perforated, advise early operative interference in nearly every form of appendicitis; but in face of the fact that about 90 per cent. of cases recover spontaneously, we cannot adopt so indiscriminate a method of treatment.

It is true that in the early stages of the disease we cannot differentiate one variety of appendicitis from another, and when first called to a case we should probably think that the symptoms would be sufficiently met by rest in bed, a moderate amount of liquid diet, the administration of a purgative by the mouth, or by rectal injection if necessary, the local application of leeches or fomentations, and a hypodermic injection of morphia for the relief of pain. These means would doubtless be sufficient for those cases which I have described as simple appendicitis and plastic appendicitis respectively, which include the great majority of cases of appendicitis and always tend to recover spontaneously.

On the other hand, this treatment would be of no avail in the rapidly perforative variety, in which the only chance of recovery is in prompt surgical interference, as early as possible after the diagnosis of perforation has been made.

Suppurative appendicitis occupies an intermediate position. There is not perhaps the urgent need for haste that exists in the rapidly perforative variety, but yet I think operative interference is equally necessary. It is perfectly true that cases of this kind may recover spontaneously by the discharge of the pus into one of the viscera or through the skin, but the chances of success are much less under these circumstances than when the patient is submitted to operation. Moreover, the patient runs the grave risk of the abscess bursting into the general peritoneal cavity.

A great deal has been written about the time of operation, but it seems to me that this only depends on the time at which suppuration is diagnosed. This may be during the first week, but is more common during the second; but whichever it may be, operation should be proceeded with as soon as the presence of pus is clearly indicated. In actual practice we must meet with cases in which we are in doubt as to whether pus is present or not. Speaking generally, there is no great harm in delaying operation under these circumstances, if the chances are as much against, as for, the presence of pus; but if we find at this time that pain is increasing, with a rapid pulse, continued elevation of temperature, and tympanites, then it would be better to operate and so endeavour to discover the position of the probable deep-seated suppuration. When I use the term "operate," I mean of course by an exploratory incision—the use of the aspirator through the abdominal wall for diagnostic purposes being generally, and very properly, condemned.

Cases of appendicitis in which the symptoms recur after a definite recovery from the former attack must be treated in accordance with the nature of the present illness, which may not be followed by any further attacks; but in relapsing appendicitis we know that the exacerbations will be repeated unless we operate, although, as I have already indicated, we operate more because of the condition of chronic invalidism than from any immediate danger to the patient. I cannot do better than quote from a recent article by Mr. Treves[1] as to the circumstances which he thinks would justify an operation in these cases. He says that in all the cases with which he has dealt, "one or other of the subjoined conditions has been present:

(1) "The attacks have been very numerous.

(2) "The attacks are increasing in frequency and severity.

(3) "The last attack has been so severe as to place the patient's life in considerable danger.

(4) "The constant relapses have reduced the patient to the condition of a chronic invalid, and have rendered him unfit to follow any occupation.

(5) "Owing to the persistence of certain local symptoms during the quiescent period, there is a probability that a collection of pus exists in or about the appendix."

He says, moreover, that he has never operated in any case in which he has not been able to make out the enlarged

[1] *Brit. M. J.*, 1893, vol. i., p. 835.

appendix still in evidence after the acute symptoms have passed away. It is, of course, well known that Mr. Treves has advocated operative procedures during the quiescent period, after the acute symptoms have subsided; and this is agreed to by most surgeons, as it is preferable not to operate during an acute inflammatory condition unless the urgency of the symptoms demands it.

To sum up, I would treat simple and plastic appendicitis on an expectant plan; but would perform abdominal section:

(1) In suppurative cases.

(2) In cases where there is good ground for *suspecting* deep suppuration.

(3) In the relapsing form.

(4) In rapidly perforative cases.

These, then, are the main principles of treatment of the various forms of appendicitis.

The all-important point is, to decide whether an operation is necessary or not, and our difficulty and anxiety in arriving at a definite conclusion on this matter are often greatly increased by the fact that we may not be called in until the prolonged administration of morphia or opium has obscured the symptoms.

It now only remains for us to consider a few operative details.

Speaking generally, the best incision is an oblique one placed at right angles to an imaginary line drawn from the right anterior superior iliac spine to the umbilicus. The incision should be two to four inches long, according to circumstances, placed about two or three inches internal to the iliac spine, and its centre corresponding to the imaginary line referred to.

It will not, however, be best to adopt this incision in all cases, especially suppurative ones. In these our incision should be placed over the seat of suppuration, not only because the pus is thus evacuated by the shortest route, but because we are more likely by this means to avoid opening, and perhaps infecting, the general peritoneal cavity. This latter point is most important, and it is chiefly for this reason that incisions in the mid-line or in the right linea semilunaris are not advisable. When adhesions are present we must exercise great care in the division of the peritoneum, or we may easily wound the subjacent intestine—indeed, in some cases this can scarcely be avoided,—but in cases of relapsing appendicitis, where adhesion of the intestines to the abdominal wall is not uncommon, Treves advises that the danger of wounding the gut is best prevented by opening the abdominal cavity beyond the diseased area where there are no adhesions.

Then comes the important question as to whether the appendix shall or shall not be removed? In some cases this is quite impossible; but our rule should be, that the appendix should be taken away if it can be removed without increasing the danger to the patient. This will depend on the number and strength of the adhesions, and the general condition of the patient; and these can best be gauged by the surgeon operating, to whose individual judgment the decision of this point must be left. We cannot formulate rules, for it is obvious that a more lengthened time in separating the adhesions and getting at the appendix might be allowable with the patient in a good condition, whereas in another case the condition of the patient might be too serious to allow of anything more than the evacuation of the pus and the insertion of a drainage-tube. Great care must be exercised in the treatment of these adhesions; and Treves thinks they are best divided with a knife, as there is less risk of opening the intestine. I believe, however, in suppurative cases it would be far wiser not to attempt the removal of the appendix at all, than to run any great risk of opening the general peritoneal cavity in the separation of adhesions.

The appendix, when found, may be so bound down that we can only throw a ligature round it and remove the portion below. The simple ligature applied in this way has been very effectual; but when it is possible and the condition of the patient allows it, I think it better to divide the peritoneum in a circular manner about half-an-inch from the cæcum, and then, having removed the appendix below, to suture the muscular and mucous walls of the stump, which is then invaginated into the cæcum and the divided peritoneum stitched over it. In this way a fæcal fistula is less likely to occur.

Any scybalon that may have escaped from the appendix should be removed, and in suppurating cases the cavity should be gently irrigated with warm boracic lotion.

In the rapidly perforative variety where there is a general infection of the peritoneum, the whole abdominal cavity should be flushed out with boracic lotion or boiled water. This is the only method which holds out any chance of success, although, so far, I believe a case reported by McBurney is the only one which has ended in recovery from this very fatal form of the affection.

A rubber tube should be inserted in the suppurative and rapidly perforative varieties, but in relapsing appendicitis neither flushing nor drainage is required as a rule.

I have now completed the task which the Council of the Association was kind enough to ask me to undertake, and which I accepted not only with a due sense of my responsibility, but with the gratifying knowledge that the doubts and difficulties incidental to the diagnosis and treatment of the disease under our consideration would be made less by the discussion which is about to follow.

---

